# Supplementary material for: Rhododendrin-Induced RNF146 Expression via Estrogen Receptor β Activation is Cytoprotective Against 6-OHDA-Induced Oxidative Stress
Source: Int J Mol Sci. 2019 Apr 10;20(7):1772. doi: 10.3390/ijms20071772 (PMC6479468; doi:10.3390/ijms20071772)
Supplement: Supplementary file 1 [file ijms-20-01772-s001.pdf]

International Journal of
Molecular Sciences

SUPPLEMENTAL INFORMATION

Rhododendrin-induced RNF146 expression via estrogen receptor β activation is cytoprotective against 6-OHDA induced oxidative stress

Hyojung Kim, Jisoo Park, HyunHee Leem, MyoungLae Cho, Jin-Ha Yoon, Han-Joo Maeng, and Yunjong Lee

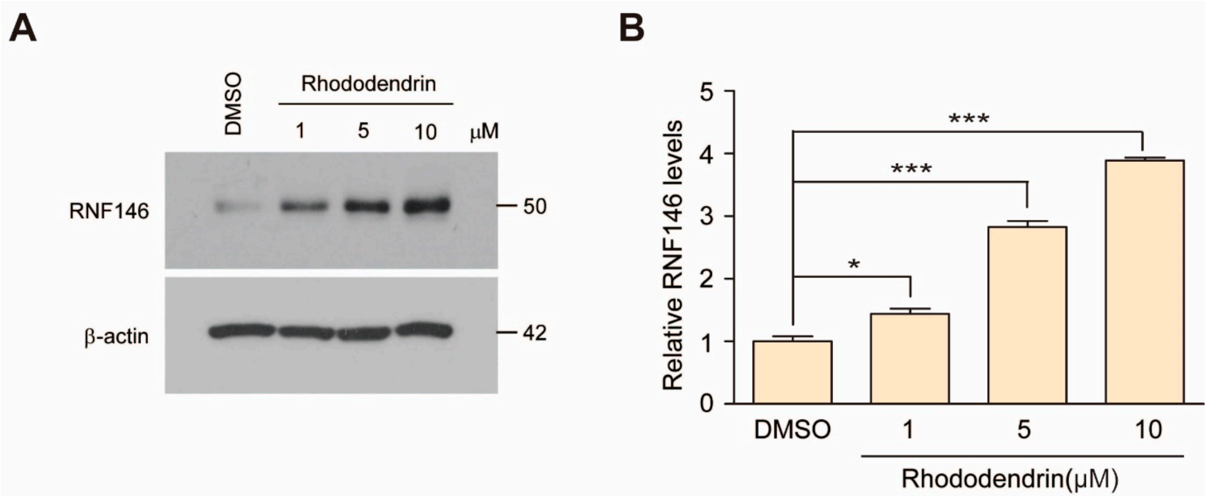

Supplementary Figure S1. Rhododendrin induced RNF146 expression in SH-SY5Y cells

(A) Representative Western blot showing RNF146 expression in SH-SY5Y cells treated for 60 h with the indicated concentrations of rhododendrin (1, 5, 10 μM). β-actin served as a loading control.

(B) Quantification of relative RNF146 protein levels normalized to that of β-actin in SH-SY5Y cells treated for 60 h with 1, 5, and 10 μM of the rhododendrin (n = 3 per group).

Data are expressed as mean ± SEM. *P < 0.05 and ***P < 0.001, ANOVA test followed by Tukey's post-hoc analysis.

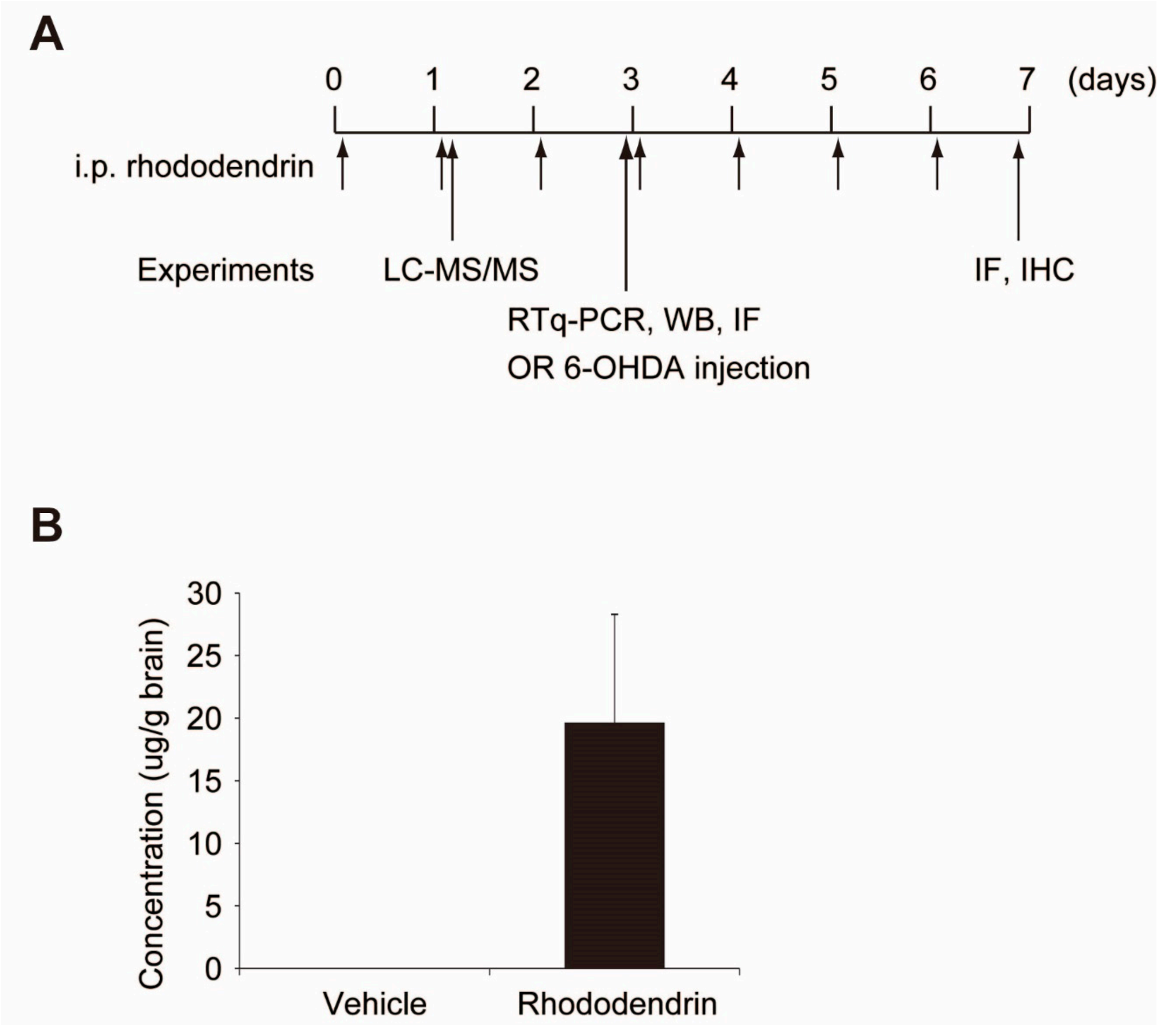

Supplementary Figure S2. *In vivo* experimental schedule and rhododendrin brain penetration.

(A) Schematic diagram depicting experimental schedule and rhododendrin i.p. administration for *in vivo* study. LC-MS/MS, high pressure liquid chromatography; WB, western blot; IF, immunofluorescence; IHC, immunohistochemistry.

(B) Quantification of brain concentration of rhododendrin in mice administered with 10 mg/kg rhododendrin i.p. (n = 3 per group) determined by LC-MS/MS. Mice were given i.p. injection of rhododendrin daily for two days, and brains were extracted 20 minutes following the second injection of rhododendrin.

Data are expressed as mean ± SEM.

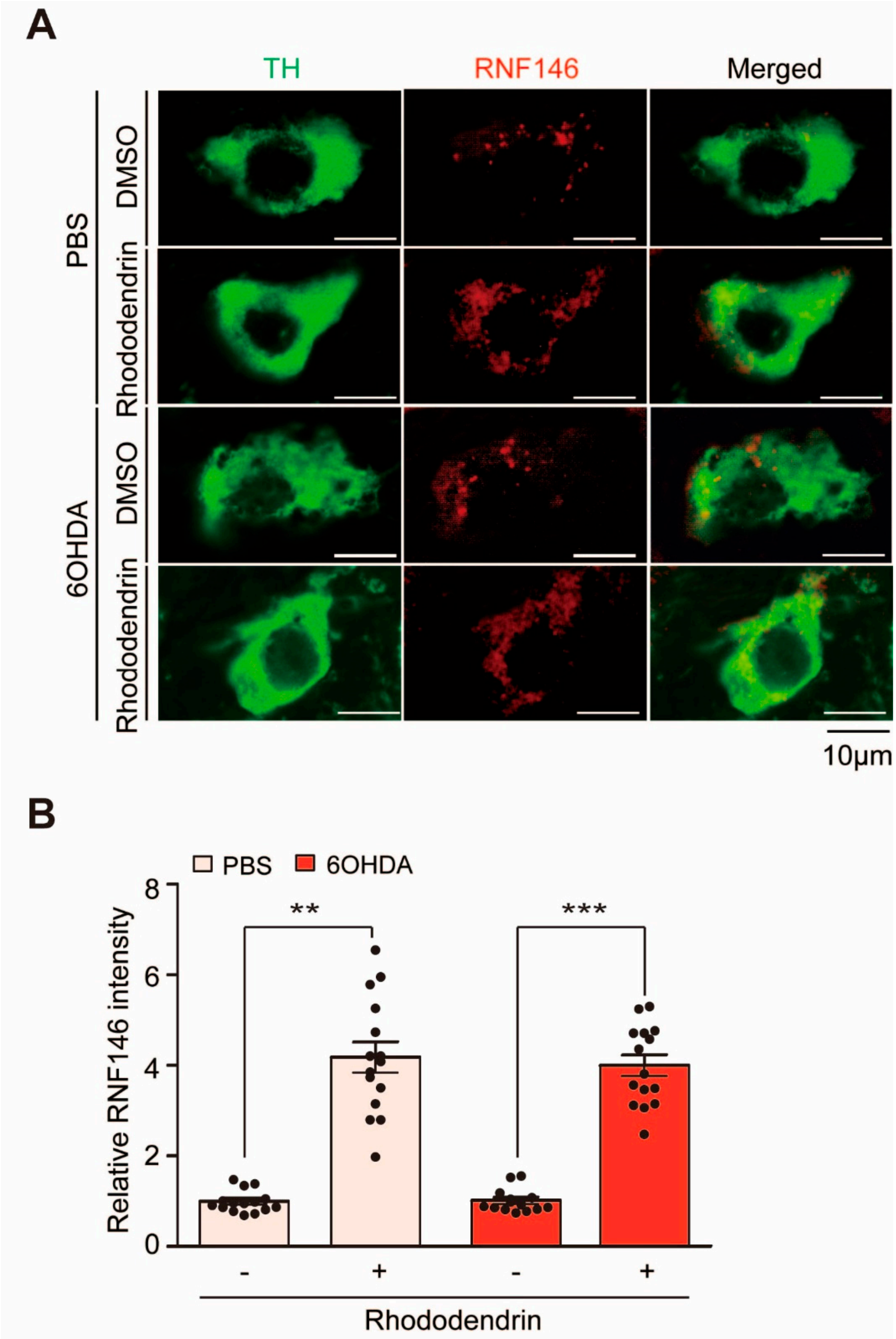

Supplementary Figure S3. RNF146 expression in dopaminergic neurons in response to rhododendrin treatment *in vivo*

(**A**) Representative confocal immunofluorescence images of TH (green) and RNF146 (red) expression using the indicated antibodies in ventral midbrain sections from 3-month-old mice treated with rhododendrin or DMSO for 7 days followed by intrastriatal 6-OHDA injection (8 ug, 4 days).

(**B**) Relative expression levels of RNF146 in TH-positive dopaminergic neurons in the indicated experimental groups as normalized to the DMSO control group (n = 15 cells per each group from three mice).

Data are expressed as mean ± SEM. ***$P < 0.001$, ANOVA test followed by Tukey's post-hoc analysis.
